# Effect of soil storage at 4 °C on the calorespirometric measurements of soil microbial metabolism

**DOI:** 10.3934/microbiol.2017.4.762

**Published:** 2017-09-11

**Authors:** Nieves Barros, Sergio Feijoo, César Pérez-Cruzado, Lee D. Hansen

**Affiliations:** 1 Department of Applied Physics, University of Santiago de Compostela, Santiago de Compostela 15782, Spain; 2 Department of Agroforestry Engineering, University of Santiago de Compostela, Lugo 27002, Spain; 3 Department of Chemistry and Biochemistry, Brigham Young University, Provo, UT 84602, USA

**Keywords:** soil, storage, heat rate, CO_2_ rate, calorespirometric ratio, microbial metabolism

## Abstract

Soil samples must usually be stored for a time between collection and measurements of microbial metabolic properties. However, little is known about the influence of storage conditions on microbial metabolism when studied by calorespirometry. Calorespirometry measures the heat rate and the CO_2_ rate of microbial metabolism, where the ratio of heat and CO_2_ released, the calorespirometric ratio, informs about the nature of substrates being used by microorganisms. Application to soil microbiology is very recent, and little is known about the influence of the common soil preparation practices between collection and analysis on the calorespirometric measurements. For these reasons, the effect of storage at 4 °C on the microbial metabolism was determined by calorespirometry. Results show CO_2_ production rate decreases with storage time while the evolution of metabolic heat rate is more stable. The calorespirometric ratio increases with storage time in soil samples with organic matter characterized by lower carbohydrate contribution to the total carbon and higher aromaticity and is unaffected in soil samples with lower carbohydrates in the organic matter and higher aromaticity. Therefore, the calorespirometric ratio values may vary for the same soil sample, such that the soil organic matter properties, as well as the time stored at 4 °C, must be considered in interpreting calorespirometric data on soils.

## 1. Introduction

One of the key aspects to study soil microbial metabolism (SMM) is the treatment of soil samples after collection and before measurement in the laboratory. Soil microbiology involves many types of measurements to study soil microbial community and soil microbial functions, with different responses to the preliminary sample treatments involving fresh or frozen soil samples [1],[2],[3]. It is generally accepted that microbial studies on soil must be done with fresh samples, but that can be difficult to achieve due to practical reasons such as a long distance between the sampling places and the laboratory where measurements will be done, sampling restricted to a short period of the year, and so on. The reality is that different features make necessary to store the soil samples before the microbial measurements. In that case and due to the high heterogeneity of soils, samples from different locations must be treated in the same way if comparative studies are to be done. Therefore, one of the main dilemmas arising in this context is how best to store samples between collection and analysis [4].

When it is not possible to work with fresh samples, frozen samples are recommended for studies of microbial community structure by molecular methods [5] but metabolic functions of microorganisms from different environments may react differently to freezing and thawing. Although Stenberg et al [6] recommended freezing when soils are stored before analysis, some authors report that microbial activities can be substantially altered in defrosted soils [1],[7]. The critical step in processing frozen soils is thawing, which can be avoided by refrigeration at 4 °C, but even in this case, changes in soils cannot be excluded [1]. In general, storage at 4 °C and –20 °C are reported to have the least impact on microbial biomass and enzymatic activities [4]. Nevertheless the effect of storage is variable and depends on the soil type and kind of measurement. Therefore, any innovation to apply in soil microbiology, involving measurement of microbial community functions, microbial community activity or microbial community structure, should consider the effect of the soil storage on the methodology, to provide the best practices for soil storage.

Calorimetry is one of the methods applied in soil microbiology to monitor SMM by the direct measurement of the heat rate, *Rq*, which measures the rate at which microorganisms degrade the soil organic matter (SOM) [8],[9]. It is a useful tool to study the reaction of soil microorganisms to external carbon, C, sources [10], the sensitivity of the microbial metabolic heat rate, *Rq*, to increasing temperature [11],[12], and the carbon conversion efficiency of microbial growth reactions by different thermodynamic models that could inform about the carbon sequestration capacity of soil microorganisms [13],[14]. In the last years, calorimetry has been adapted as a calorespirometric procedure, to measure both, the CO_2_ rate, *R*_CO2_, and the *Rq* of SMM. The ratio of *Rq* to the *R*_CO2_ is the calorespirometric ratio of SMM, CR, a metabolic indicator that has been applied to many living systems [15] (cells, plants, insects, microorganisms and so on) and that informs about the nature of substrates being metabolized when quantified at metabolic steady state conditions, that is when applied to microbial reactions that do not involve microbial C net gain. This information can be useful when applied to microbial systems degrading complex substrates as soils. For these reasons, it has been started to be applied in soil science, but procedures to quantify CR from soil samples are very recent [16],[17],[18]. Initial results from different soil samples showed that CR varied a great deal among soils [16] making that it was necessary to search for principal causes responsible for CR variations. With that goal in mind, recent papers report different factors influencing the CR of SMM, including previous management of soil for laboratory analysis, such as mechanical sieving, and soil water content [19], as well as the response to increasing temperature [20], settling the effect on the CR.

By definition, CR should be related to the nature of substrates being metabolized by soil microorganisms when measured in soil samples at a microbial metabolic steady state, by the following equation:

$$\gamma_{S}=4\;\left(1-\frac{CR}{455}\right)$$ (1)

where *γ*_S_ is the oxidation state of the C being oxidized to CO_2_ by the soil microorganisms and 455 is the constant from Thornton's rule [19],[21]. Therefore, different CR values should be obtained when measured for soils with different SOM properties. As an example, it is reported a CR value of about –460 kJ mol^−1^ for respiration of carbohydrates, and higher or lower CR values would indicate metabolism of substrates more reduced or more oxidized than carbohydrates respectively [21]. In order to verify the applicability of equation 1 in soil research, CR has been determined in different soil chronosequences characterized by SOM evolving towards a more recalcitrant state [19],[22] and in different soil ecosystems [17] to evaluate the role of the SOM nature on CR values. These results launched robust evidences that equation 1 could define CR values for SMM but also showed that some soils, with presumably more labile organic matter, could have more variable CR values than soils with more recalcitrant or stable organic matter.

One of the factors affecting CR changes in samples with labile SOM could be the storage conditions prior microbial calorespirometric measurements, which in fact, is one of the factors involving soil sample preparation that had not been studied yet. Most of the calorimetric measurements on soils have been done with soils stored at 4 °C for about 3 to 6 months after sampling. However, the impact of sample storage on calorimetric measurements of SMM has been little explored with no evidence of how the time of storage at 4 °C effects CR values. The only study available reports a diminished metabolic heat rate after 6 months of storage at 4 °C in soil samples amended with glucose [23].

This work evaluates storage consequences on the heat rate, CO_2_ production rate, and CR of soil microbial metabolism when measured by calorespirometry, for soils originating from Pasture, young forests and mature forests, in order to evaluate the extent of the effect of soil storage at 4 °C on CR values in soils with different SOM properties.

## 2. Materials and Methods

### 2.1. Soil samples

Soil samples used for this work were cambisols collected in the northwest of Spain (Castro del Rey 43° 12′31″ N 7° 24′ 1″ W) in a well-known chronosequence network representing the conversion of pastures to forests with *Pinus radiata*. Four different sampling sites were previously established based on the chemical and thermal properties of the soils. Soil samples for this study were selected to represent labile and recalcitrant soil organic matter (SOM) based on previously determined chemical and thermal properties [24]. A plot was established in each of the four sampling sites to take six soil subsamples with a steel corer at 0–10 cm, after removal of surface litter, to be combined into one sample per site as reported in previous papers [19],[24]. Samples from a “Pasture” and a young afforested forest (“P10”, 10 years since afforestation) were chosen as representing more labile SOM. Samples from a mature afforested forest site (“P30”, 30 years since afforestation) and a forest reference without previous history of afforestation (“FR”) were chosen to represent more recalcitrant SOM.

Recalcitrance and lability of the soil samples were assumed based on the soil chemical and thermal properties determined by ^13^C CPMAS and thermal analysis, as explained in detail in a previous paper [24]. Thermal stability of samples was studied by differential scanning calorimetry and quantified by the T50-DSC, defined as the temperature at which 50% of the energy of the substrate is released. It is assumed that higher T50-DSC involves higher thermal stability of SOM that could be associated with higher SOM recalcitrance [12],[25].

### 2.2. Soil sample treatment

To replicate samples preparation in previous studies, samples from each sampling site were sieved (2 mm) and air dried for 3 days at room temperature (20 °C) to remove excess water. Drying and rewetting can strongly modify the soil microbial community if soils are dried for 14 days under stress conditions [4],[26] but these conditions were not applied here or in earlier calorespirometric studies. After drying, 10 g of soil from each sampling location was prepared for immediate calorimetric measurements and considered as a fresh sample. The remainder of each sample from each sampling site was kept inside polyethylene bags, and calorimetric measurements were done after 1 and 3 months of storage at 4 °C, the typical time required for processing a representative number of samples by calorespirometry.

Samples were prepared for measurement by adding sterile deionized water to adjust water content to 60% of water holding capacity (WHC) and equilibrating at 25 °C during 4–5 days to stabilize the soil after water amendment. Equilibration was done inside polyethylene bags with an open water container to prevent drying. The duration of this stabilization was tracked by calorimetry before starting the experiments.

Soil subsamples stored at 4 °C for 1 and 3 months were pre-equilibrated from 4 to 25 °C by holding the samples for 24 hours at 25 °C inside polyethylene bags. After this pre-equilibration, soil moisture was adjusted to 60% of WHC by the same procedure applied to the fresh samples.

### 2.3. Calorespirometric measurements

Calorespirometric measurements were done with a six channel TAM III calorimeter (TA Instruments, Lindon, UT) following the same procedure explained in previous papers [19]. In each calorimetric measurement, six aliquots of 1 g each from a 10 g subsample were sealed in 4 ml stainless steel ampoules and placed in the calorimeter. A small vial with 0.4 M NaOH was introduced into three of the ampoules to measure the sum of the metabolic heat and CO_2_ rates. Measurements were done at 25 °C under isothermal conditions. The procedure for the simultaneous measurements of heat and CO_2_ is explained in detail in previous papers [19],[22]. Calorespirometric measurement with 6 ampoules of each sample takes 48 hours, yielding triplicates of the CO_2_ and heat rates (n = 3 ± SD) for each soil. Because heat and CO_2_ rates usually deplete during the 48 hours measurement time and are not constant, the measured rates were averaged over a 22 hour time period excluding the initial equilibration period, by integrating the heat and CO_2_ rates over the same time period of 22 hours, to give the heat rate, *Rq*, in milijoules per gram of soil per hour, and the CO_2_ rate, *R*_CO2_, in micromole CO_2_ per gram of soil per hour, a common measure of how fast SOM is degraded by microbial action [25]. CR ratio is determined by the ratio of the *Rq*/*R*_CO2_ values at steady state soil microbial metabolic rates, with no gains in microbial biomass during the measurement. *Rq* and *R*_CO2_ values were normalized to the C content of the soil samples too, to obtain the heat and CO_2_ rates per soil C gram, a measurement of soil biological stability [22],[25] in order to compare the biological stability of these soils and to see how storage may affect to these values in comparative studies. It is assumed higher biological stability in soils with lower heat and CO_2_ rates per gram of soil C.

### 2.4. Statistical analysis

The significance of differences in mean values for heat and CO_2_ rates from each soil sample after storing at different times was tested by a t-test using the samples considered as fresh as a reference (*P* < 0.05; n = 12). Heat and CO_2_ rates from different sites was tested by one way ANOVA (*P* < 0.05, n = 9) by considering the different sites as levels of the studied factor. Normality and homogeneity of variances of the data were evaluated by the Shapiro-Wilk and Bartlett tests, respectively. Post-hoc differences among the levels of the factor were evaluated by the Tukey HSD test. All statistical analyses were performed by R statistical software [27].

## 3. Results

Table 1 gives the chemical and thermal properties of the samples. SOM from the Pasture and young forest (P10) sites have a higher ratio of carbohydrate to total C, are less aromatic, and less thermally stable (lower T50-DSC values) than SOM from the mature forest sites (P30 and FR) (Table 1).

**Table 1.** Chemical and thermal properties of the samples. A/O-A is the Alkyl-C to O-Alkyl-C ratio. Aro-C is the percentage contribution of the aromatic carbon to total carbon. Alkyl-C is the percentage contribution of aliphatic carbon. O-alkyl C is the percentage contribution of carbohydrates and Carbonyl the contribution of carbonyl groups. T50-DSC is the temperature at which 50 % of the energy of the OM is released, and it is considered as an index of thermal stability. Higher temperature involves higher stable material.

Samples	Pasture	P10	P30	FR
Elemental Analysis				
Carbon content, %	8.0 ± 0.2	2.7 ± 0.2	9.4 ± 0.6	11.5 ± 0.3
Nitrogen content, %	0.44 ± 0.01	0.22 ± 0.01	0.49 ± 0.06	0.45 ± 0.02
C/N	18 ± 1	12 ± 1	19 ± 1	26 ± 1
pH	4.44	3.98	3.56	3.12
Thermal Analysis				
T50-DSC, °C	336 ± 2^a^	335 ± 1^a^	348 ± 1^a^	366 ± 2^a^

^13^C CPMAS^a^				
Samples	Pasture	P10	P30	FR
Alkyl-C, %	24	25	26	26
O-Alkyl-C, %	52	43	38	40
Aro-C, %	19	19	27	25
Carbonyl, %	5	13	9	9
A/O-A	0.46	0.58	0.68	0.65
Aromaticity	0.20	0.22	0.30	0.28

^a^Thermal and ^13^C-CPMAS^a^ data published in Pérez-Cruzado et al. 2014 [17] with permission of Springer Science + Business Media.

**Figure 1.**
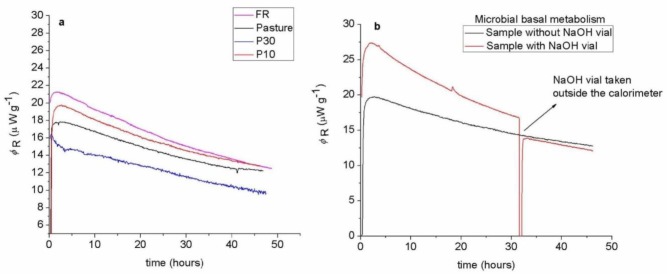
(a) Examples of power-time plots representing the heat rate (*φ*_R_) of soil microbial metabolism in the samples recorded by calorimetry under metabolic steady state conditions. The heat rate, measured directly in microwatts by isothermal calorimetry at 25 °C, declines with time, and shows no increase as would occur with microbial growth. Therefore, there is no increase in living microbial biomass during the measurement. (b) Shows the power-time plots measured by calorespirometry with a NaOH vial in the calorimetric ampoule. The arrow shows where the NaOH vial was taken out of the calorimeter to check the reproducibility of the microbial metabolism in the same soil. *Rq* and *R_CO2_* are determined by integration of these power-time plots over 22 hours and dividing by the time.

Heat and CO_2_ rates from microbial metabolism recorded over 48 hour on fresh and stored soils were stable or declined slightly, indicating there was no net microbial growth during the measurement (Figure 1).

**Figure 2.**
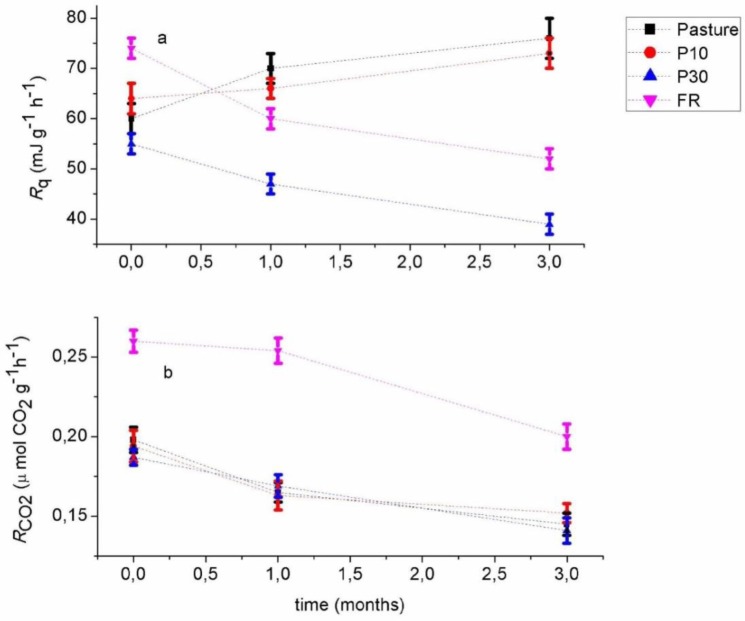
Heat (*R*q) rates (a) and CO_2_ (*R_CO2_*) rates (b) versus the sample storage time.

Figure 2 shows how the quantitative heat (*Rq*, 2a) and CO_2_ rates (*R*_CO2_, 2b) evolve over storage time. *R_CO2_* tended to decrease in all samples as storage time increased. t-test results showed that these differences attached to the time of storage were significant after 3 months of storage at the 0.05 significance level. Samples from the Pasture and young forest site (P10) showed a significant decrease (*P* < 0.05) in *R_CO2_* of –17 and –20%, respectively. Samples from the mature forest sites (P30 and FR) did not show a significant decrease in *R_CO2_* after the first month of storage, but did show a significant decrease (–25 and –23%, respectively) after 3 months of storage. ANOVA indicated *R*_CO2_ values from the different samples sites where significantly different (Fvalue16.29; Prob > F 1.30e–6). Tukey's test revealed that the forest reference (FR) had a significantly higher respiration rate (*P* < 0.05).

Trends in *Rq* with storage time depended on the soil sites. *Rq* increased with time of storage in the Pasture and P10 samples (27 and 13%, respectively after 3 months) and decreased with time of storage in the P30 and FR samples (–30 and –31%, respectively after 3 months) but the t-test revealed that these differences were not significant in the pasture and P10 sample (*P* > 0.05). ANOVA and Tukey's test showed *Rq* values were significantly different among the sites (Fvalue 14.49; Prob > F 3.24e–6) but not significantly different between the Pasture and the young forest site (P10) at the 0.05 level.

**Figure 3.**
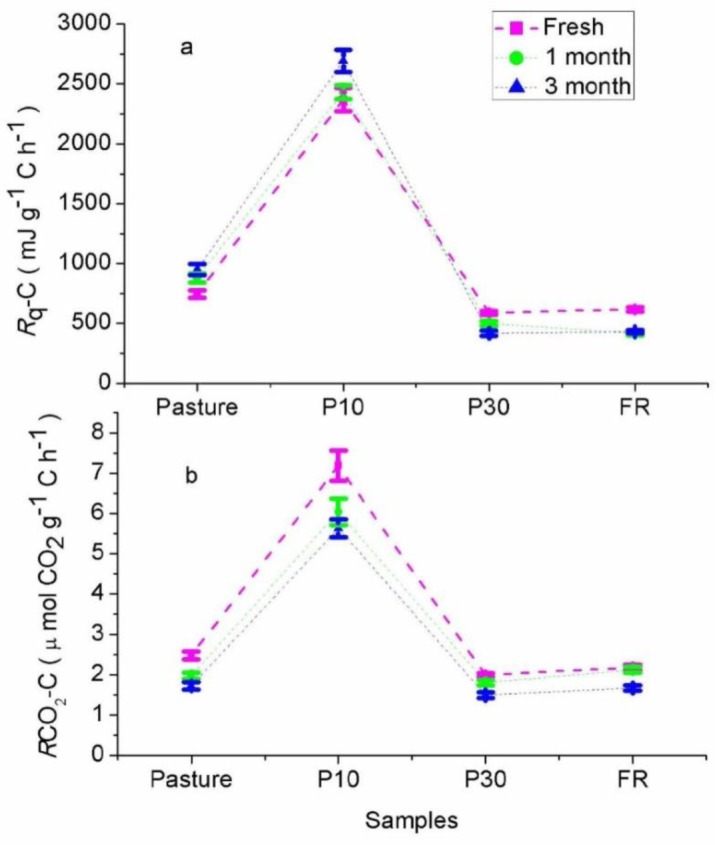
Heat (*R*q) rates (a) and CO_2_ (*R_CO2_*) rates (b) normalized to the sample C content.

Figure 3 shows the heat and CO_2_ rates (Figure 3a and Figure 3b respectively) normalized to the C content of the samples to compare the soil biological stability. Soils from the Pasture and young forest site had higher heat and CO_2_ rates per unit of C than the samples from the mature forest sites, being remarkable higher in the young forest (P10) than in the mature forest. ANOVA yielded significant differences among sites (Prob > F 1.83e–5; Prob > F 1.46e–5 for the CO_2_ and heat rates respectively). Tukey's test showed means of the heat and CO_2_ rates normalized to the C content were not significantly different between P30 and FR samples representing mature forest sites (*P* > 0.05).

**Figure 4.**
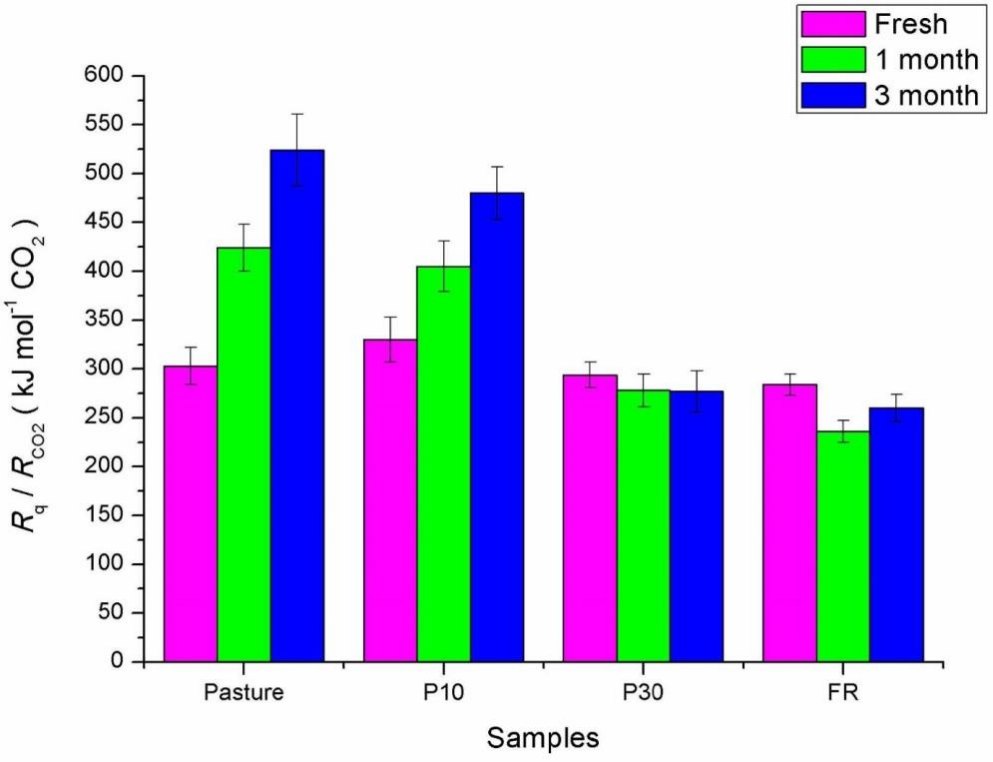
Calorespirometric ratios (CR) obtained at different storage times.

Figure 4 shows how the calorespirometric ratio (CR) evolves over storage time. ANOVA yielded significant differences of CR values among sampling sites (Fvalue 13.45; prob > F 7.57e–6). Soil samples from Pasture and young forest (P10) show increasing CR with storage time. Tukey's test revealed that values in samples from mature forests (P30 and FR) were not significantly different (*P* > 0.05). Comparison of CR average values and SD suggest no remarkable changes of CR values in P30 sand FR samples with storage time, and that these values did not differ in all fresh samples.

The observed evolution of CR with storage time in some of the samples may be indicating a change in the nature of SOM during storage. These changes can be evaluated by the application of equation 1 that defines CR. As *γ*_S_ for carbohydrates is 0, CR values higher than 455 would be obtained for negative *γ*_S_ values, and CR values lower than 455 for positive values of *γ*_S_, that is CR decreases as the organic substrate degraded by soil microorganisms is more oxidized. Based on equation 1, an average carbon oxidation number of +1.3 ± 0.2 is determined for the substrate being oxidized by soil microorganisms in all fresh samples. The average *γ*_S_ values for P30 and FR samples is +1.6 ± 0.2 which does not significantly change with storage time. In the Pasture, *γ_S_* decreases to +0.2 after one month and to –0.6 after three months. In P10, *γ_S_* decreases to +0.4 after one month and to –0.2 after three months. Therefore the observed evolution of CR values in Figure 4 suggests changes in the SOM nature along the measurement affecting the Pasture and P10 samples.

## 4. Discussion

Results evidence how metabolic rates may give different responses to the same soil treatment. Increasing storage time at 4 °C caused a general trend of soil microbial respiration to decrease in all soil samples while the metabolic heat rate was more insensitive to the storage conditions and only two of the samples showed the same trend of the CO_2_ rates to decrease after 3 months of storage. When both, CO_2_ and heat rates, are normalized to the C content of the soil to evaluate the biological stability (quantity of heat and CO_2_ released per unit of soil C) the evolution of the SOM biological stability among soil samples is not altered by soil storage and keep the same trend reported in previous papers [19],[24], showing that samples from mature forest sites (P30 and FR with lower heat and CO_2_ rate values per unit of soil C than the other samples, P10 and Pasture) have higher biological stability than the young forest site independently of the time of storage along 3 months. Therefore, under the storage conditions used, the evolution of the CO_2_ and heat rates per unit of soil C, would not affect the conclusions in comparative studies if all samples are stored during the same time and at the same conditions. However, CO_2_ production rates (micromol CO_2_ per gram of soil and hour) decreased significantly by –13 to –31% after three months of storage in all samples, an effect that should be taken into account if the goal of the research is just quantitative determinations of the CO_2_ rates.

By the monitoring of *R*_CO2_ and *R*q rates, we can only measure how fast or slow SOM is degraded by microbial action and how those rates evolve with the time of storage, without specific mechanisms. The introduction of new metabolic indicators like CR can give additional information about how the soil biochemistry was affected by the storage conditions.

CR values were similar in all fresh samples, and they suggest microbial degradation of substrates more oxidized than carbohydrates, indicating that sieving may release substrates at higher oxidation state than carbohydrates that could temporarily alter the soil microbial biochemistry [28]. CR values for pasture and P10 samples increased with storage time, while CR values for mature forests were unaffected. As a consequence, the degree of reduction (turn of *γ_S_* to negative values) of the substrates being metabolized by soil microorganisms, obtained by equation 1, increased with time of storage at 4 °C in the Pasture and P10 samples. This trend can be attributed to the depletion of carbohydrates in refrigerated soils due to ongoing microbial activity [14],[29]. In fact, CR values obtained for the Pasture and P10 soil samples after 1 month at 4 °C indicate respiration of carbohydrates in agreement with the low temperature activities of enzymes such as β-glucosidase that hydrolyzes cellobiose to glucose [4]. After 3 months, CR values indicate substrates being metabolized are more reduced than carbohydrates and are compatible with the biodegradation of proteins and/or lignin [19],[21].

CR values in the soil samples from mature forest sites (P30 and FR) were insensitive to time of storage. The CR values in these samples indicate a microbial metabolism based on a mix of decarboxylation and oxidation reactions [19]. This is in agreement with recent papers reporting that old, stable, organic carbon associated with mature forest sites supports microbial communities less adapted to complex polymers but better adapted to using readily oxidizable, although energetically less rewarding, substrates [30].

The observed different trends in CR values among these samples as time of storage increases could be attributed to the different SOM nature. The samples from the Pasture and young forest site (P10) have SOM with higher contribution of carbohydrates to total C, lower aromaticity and lower thermal stability than SOM from the mature forest sites (P30, FR). Based on these results, samples with high contribution of carbohydrates in their SOM composition, continue to degrade the carbohydrate fraction at 4 °C, and for this reason the same soil samples may yield different CR values as the SOM turns to a more reduced state, while the biochemistry of SOM with lower contribution of carbohydrates and higher thermal stability is more stable and insensitive to storage at 4 °C at least during 3 months.

## 5. Conclusions

Storage at 4 °C does not alter the evolution of the CO_2_ and heat rates per unit soil C from younger to mature forest soils but affects soil microbial biochemistry, particularly in the soil samples with more labile SOM.

CR values show that samples with labile SOM have higher microbial metabolic diversity, indicating a microbial metabolism capable of degrading substrates more and less reduced than carbohydrates, and it is more sensitive to storage conditions than microbial metabolic diversity in stable SOM. For this reason, the same soil sample could yield different CR values for calorespirometric measurements run at distinct times.

A general procedure assessing sample storage for soil microbiological measurements is probably not achievable or desirable because different technological applications require different storage conditions in order to obtain the best results. Optimum sample storage conditions depend on the method to be applied, on the goal of the research, and now, on SOM nature if soil microbial metabolism is studied by calorespirometry. Since sieving is also applied to all samples and releases substrates, thus altering the natural soil biochemistry, stabilization of samples for at least one month, particularly when working with labile OM, could provide for more reliable measurements of the metabolic properties than fresh samples when measured by calorespirometry.
